# Modifying modularity: aerobic exercise improves functional connectivity in breast cancer survivors

**DOI:** 10.3389/fcogn.2024.1323438

**Published:** 2024-02-02

**Authors:** Lindsey L. Page, Abi Heller-Wight, Connor J. Phipps, Ann M. Berger, Elizabeth C. Reed, David E. Warren, Diane K. Ehlers

**Affiliations:** ^1^Department of Neurological Sciences, University of Nebraska Medical Center, Omaha, NE, United States; ^2^College of Nursing, University of Nebraska Medical Center, Omaha, NE, United States; ^3^Department of Internal Medicine, University of Nebraska Medical Center, Omaha, NE, United States; ^4^Mayo Clinic Arizona, Scottsdale, AZ, United States

**Keywords:** exercise, breast cancer, magnetic resonance imaging, executive functions, aging

## Abstract

**Introduction:**

Aerobic exercise has been shown to improve cancer-associated cognitive decline (CACD) in breast cancer survivors (BCS), and recent findings suggest that one mechanism by which exercise may reduce cognitive decline is through alteration of the brain's functional organization. Many cognitive abilities and measures of functional brain organization change with age and disease, typically reflected in cognitive decline and reduced differentiation of brain networks, or “modularity.” Although previous research has identified associations between lifestyle interventions, such as exercise, and increased modularity, no studies have examined these relationships in cancer populations. The primary aim of this study was to investigate the preliminary effects of a 12-week aerobic exercise program on changes in brain network modularity in BCS. As a secondary aim, we explored correlations between changes in modularity with moderate-to-vigorous physical activity (MVPA) and cognitive function. Data were exploratory and used for hypothesis generation for a future, larger study.

**Methods:**

Participants included a subsample of 10 BCS (*M* age = 65.9 ± 9.3 years) from a larger pilot study (*N* = 30 BCS) who were randomized to a 12-week aerobic exercise program (AE) or usual care (UC). The present study collected brain magnetic resonance imaging, Actigraph accelerometry, and cognitive task performance at baseline and 3-month follow-up (i.e., post-intervention; *n* = 4 AE, *n* = 6 UC). Intervention effects on modularity, MVPA, and cognition were quantified as magnitude of change between groups (Cohen's d). Changes in modularity were further explored via paired *t*-tests within groups. Associations between changes in modularity, MVPA, and cognitive performance were explored using Spearman's correlations.

**Results:**

The magnitude of changes in modularity between groups were small-to-moderate and favored the AE group (d = 0.23 to d = 0.67 across thresholds). Paired *t*-tests revealed a significant increase in modularity in the AE group from baseline to 3-month follow-up (*t* = 3.08, *p* = 0.03, *d* = 1.17), but not in the UC group. The correlation between changes in MVPA and changes in modularity were not statistically significant (*r* = 0.36, *p* = 0.39), and correlations between modularity and cognitive performance yielded mixed effects by cognitive domain.

**Discussion:**

Findings suggest that aerobic exercise may influence functional brain network organization and cognition in BCS. These data warrant further investigation in larger exercise trials.

## 1 Introduction

Breast cancer is the most commonly diagnosed cancer among women in the United States (Siegel et al., 2023). Although improvements in early detection and treatment have increased 5- and 10-year survival rates, survivors are often left with long-lasting, negative side effects of treatment (Ahles et al., 2012; Ahles and Root, 2018). Cancer associated cognitive decline (CACD), characterized by deteriorations in executive function and memory processes, has emerged as one of the most disruptive of these consequences (Shilling et al., 2005; Stewart et al., 2006; Jansen et al., 2011; Ahles et al., 2012; Janelsins et al., 2014; Mandelblatt et al., 2016; Ahles and Root, 2018). Previous studies have estimated that up to 78% of breast cancer survivors (BCS) report cognitive changes, and, even decades after treatment, more than 20% report persistent cognitive deficits (Koppelmans et al., 2012; Wefel and Schagen, 2012; Mandelblatt et al., 2018). The most common deficits include impairments in executive functions (i.e., top-down mental processes that regulate behavior) and working memory (i.e., ability to store, manipulate, and apply information) (Horowitz et al., 2019) that can impact long-term functioning, independence, and quality of life (Calvio et al., 2010; Vega et al., 2017; Mandelblatt et al., 2018). As the number of cancer survivors continues to increase (Siegel et al., 2021), there is growing need to understand and alleviate CACD.

As the field of CACD research has grown, accumulating evidence suggests that established neuropsychological assessments might benefit from supplementation by functional neuroimaging approaches that measure brain activity at rest or during task performance (Correa and Ahles, 2008). Although functional brain correlates of cognitive impairment in BCS have not been fully elucidated, functional MRI (fMRI) approaches have provided preliminary insights into widespread brain dysfunction as a consequence of cancer (McDonald and Saykin, 2013; Sousa et al., 2020; Mzayek et al., 2021; Phillips et al., 2022). For example, an early fMRI study found that, despite similar performance on cognitive tasks, BCS treated with chemotherapy exhibited broader spatial activation during working memory tasks, a finding that could reflect functional compensation during task performance (Ferguson et al., 2007). Additionally, more recent fMRI studies measuring properties of functional brain networks have identified patterns of altered network organization associated with reduced network efficiency, disrupted network communication, and reduced connectivity between regions related to attention dysfunction (Miao et al., 2016; Phillips et al., 2022).

Assessment of functional brain networks, as measured with fMRI through resting state functional connectivity (rs-FC), has benefitted from the application of tools from the field of graph theory that were developed to study many types of networks. One such tool is modularity, a graph theory metric used to evaluate whole-brain functional connectivity. Modularity is a scalar value that summarizes a contrast between the number connections within and between the brain's intrinsic functional networks (Baniqued et al., 2018). This measure has been used to test for differences in functional brain organization in a variety of clinical and healthy aging populations because it is a concise quantification of functional brain organization (He and Evans, 2010; Park and Friston, 2013; Cohen and D'Esposito, 2016; Baniqued et al., 2018; Gallen and D'Esposito, 2019; Esfahlani et al., 2021). Measures of functional brain organization may have utility in populations such as BCS who experience subtle changes in cognition that are hard to detect with simple behavioral measures (Ahles and Root, 2018). Modularity has not yet been examined in cancer survivors but has the potential to elucidate underlying brain changes and aid in understanding the mechanisms involved in CACD (Sun et al., 2012; Aboud et al., 2019).

Although there is no established treatment for CACD in BCS, evidence of the neurocognitive benefits of regular exercise is promising. Preliminary studies in adult cancer populations suggest regular exercise may benefit cognition (Campbell et al., 2020); however, few interventions have measured cognitive function objectively or focused on neurocognitive function as a primary outcome (Sturgeon et al., 2023). Even fewer studies in cancer have evaluated the effects of exercise interventions on brain structure or function (Koevoets et al., 2023). Applications of aging frameworks may be useful in expanding this evidence base (Mandelblatt et al., 2013; Ehlers et al., 2016; Ahles and Root, 2018; Ahles et al., 2022). For example, studies of exercise in healthy aging populations have shown that physical exercise attenuates age-related cognitive decline, and one suggested mechanism for this effect is greater segregation of functional brain networks reflected in increased modularity (Sun et al., 2012; Aboud et al., 2019). Exercise trials in healthy aging populations have also demonstrated changes in rs-FC and linked modularity profiles with improved cognition as a function of exercise (Burdette et al., 2010; Voss et al., 2010, 2020; Ehlers et al., 2016; Li et al., 2017; Baniqued et al., 2018; Prehn et al., 2019; Stillman et al., 2020). Indeed, Voss et al. (2010) demonstrated a significant relationship between cardiovascular fitness, brain modularity, and cognitive abilities in older adults such that higher levels of fitness were associated with improved resting functional efficiency and increased brain network modularity. Subsequent studies have identified significant associations between fitness and rs-FC changes (Voss et al., 2016) and found that baseline modularity predicted improvements in executive functions among older adults who were enrolled in an aerobic exercise program (Baniqued et al., 2018). While associations of fitness and brain network organization appear to be robust and reproducible findings, thus far no studies have, to our knowledge, explored the effects of an exercise intervention on brain modularity in cancer survivors. To the extent that the cognitive declines observed in healthy aging and in BCS with CACD are the result of shared mechanisms, treatments known to improve cognition and brain health in healthy aging might also benefit cancer survivors (Mandelblatt et al., 2013; Ahles and Root, 2018; Ahles et al., 2022).

The purpose of this study was to examine the effects of a 12-week, pilot aerobic exercise intervention on whole-brain modularity in BCS. We hypothesized that BCS randomized to the exercise intervention, compared to those assigned to usual care, would exhibit increased whole brain modularity as measured through rs-FC. As this was a pilot study, the primary objective was to generate preliminary data on the utility of modularity as a novel biomarker of brain changes associated with engagement in regular physical activity in BCS. Among our secondary outcomes, we also explored correlations between changes in modularity and (1) change in moderate-to-vigorous physical activity (MVPA) and (2) change in cognitive performance on tasks measuring executive functions, working memory, and verbal memory.

## 2 Materials and methods

### 2.1 Study design and participants

Participants in this study were a subsample of BCS (*n* = 10) enrolled in a pilot randomized exercise trial (*N* = 30) who agreed to undergo brain MRI. Participants were recruited via targeted mailings to breast oncology patients at an academic medical center and private cancer center as well as flyers distributed to community organizations, social media posts, and word of mouth. Eligible individuals included adult women aged 21 years or older who (1) were diagnosed with stage I–IIIa breast cancer; (2) had completed primary treatment (i.e., surgery, chemotherapy, radiation therapy) within 3 – 24 months of screening; (3) were postmenopausal at the time of their diagnosis; (4) had no prior history of cancer, excluding non-invasive skin cancer; (5) had no history of stroke, transient ischemic attack, other neurological disorders, or brain surgery involving tissue removal; (6) scored >21 on the Telephone Interview of Cognitive Status – Modified (TICS-M); (Brandt et al., 1988) (7) self-reported, on average, fewer than 60 minutes of moderate intensity physical activity per week in the last 6 months; (8) received physicians' clearance to engage in exercise; and (9) screened for safe participation in an MRI environment (e.g., no metallic implants or claustrophobia).

Interested individuals were contacted via phone to confirm eligibility and schedule an orientation. Participants provided informed consent according to the Declaration of Helsinki before data collection began. Participants completed testing (brain imaging, physical activity monitoring, cognitive assessments) prior to randomization and after the 12-week period. Participants were randomized to the aerobic exercise program (*n* = 15) or usual care (*n* = 15) following a blocked randomization scheme. Among the women who agreed to undergo neuroimaging, 4 were randomized to aerobic exercise and 6 were randomized to usual care. This study was approved by the University of Nebraska Medical Center IRB and is registered with the National Institutes of Health (ClinicalTrials.gov) (NCT03980626).

### 2.2 Study groups

#### 2.2.1 Aerobic exercise program

**Figure 1:**
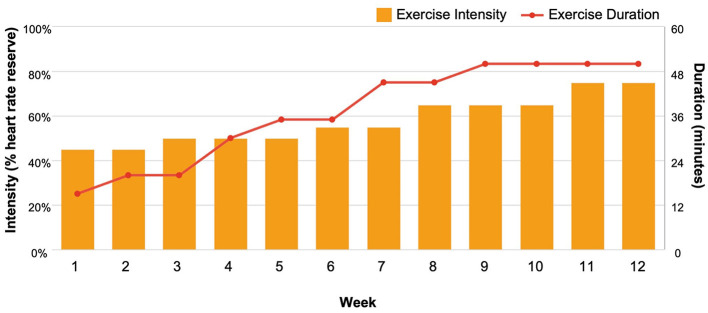
Exercise protocol.

Participants randomized to the aerobic exercise program (*n* = 4) engaged in thrice-weekly small-group or one-on-one walking sessions led by American College of Sports Medicine (ACSM) certified exercise physiologists at local fitness centers (i.e., YMCAs). The exercise protocol (Figure 1) followed ACSM guidelines for exercise in cancer survivors (Campbell et al., 2019) and was progressive such that exercise intensity and/or duration training principles were manipulated weekly to increase physical activity volume and cardiorespiratory fitness over the 12-week program. Participants' individualized exercise prescriptions began at ~45%−50% heart rate reserve for 15 min−20 min in Weeks 1–3 and progressed to 60%−75% heart rate reserve for 45 min−50 min by Week 9. Each exercise session began with a 5-min warm-up and ended with a 5-min cool-down. Across the study, *n* = 140 sessions were completed as originally designed, and n = 30 sessions were modified due to the COVID-19 pandemic (one participant completed a modified exercise program in which exercise sessions 7–36 were completed as home-based, unsupervised exercise sessions supplemented with weekly virtual exercise counseling).

#### 2.2.2 Usual care

Participants randomized to the usual care arm (*n* = 6) were asked to continue cancer care as usual. BCS assigned to this group were not discouraged from engaging in exercise; however, they did not receive information regarding physical activity behavior change, exercise prescription, or fitness levels during the 12-week period. After follow-up assessments were complete, usual care participants were offered an individualized exercise prescription and a 3-month membership to a local fitness center.

### 2.3 Measures

All outcomes were measured at baseline (prior to randomization) and 3-month follow-up (immediately following the 12-week intervention or usual care period). Participants completed brain imaging and cognitive testing in two separate appointments lasting ~1 h each. Between data collection appointments, participants were instructed to complete questionnaires and wear an accelerometer.

#### 2.3.1 Demographics and breast cancer history

Demographic information (i.e., age, race, education, income, employment status, marital status, comorbid conditions) was collected via self-report survey during baseline testing. Clinical information on breast cancer diagnosis and treatments received were obtained retrospectively using electronic medical records.

#### 2.3.2 Physical activity

Participants were asked to wear an accelerometer (Actigraph GT9X, Pensacola, FL, USA) for 7 consecutive days at baseline and 3-month follow-up to measure MVPA. Participants were advised to wear the device on their non-dominant hip during waking hours. Accelerometer data were included for analysis if the participant had 10+ hours of wear time on at least 4 of the 7 days (Troiano et al., 2008). Average daily minutes of MVPA were determined using Freedson cut points (1952+ counts/minute) (Freedson et al., 1998).

#### 2.3.3 MRI acquisition and processing

MRI data were collected using a Siemens Prisma 3 Tesla MRI system with a 32-channel head coil. All scans were performed by the MRI Research Specialist using NUMARIS/4 Syngo MR VE11C software. Any excess space within the head coil was filled with foam padding to enhance comfort and limit head motion during scanning. Participants were instructed to remain as still as possible for all scans. The MRI protocol was adapted from the Lifespan Human Connectome Project Aging (HCP-A) (Harms et al., 2018). T1- and T2-weighted anatomical whole brain scans were first acquired. T1-weighted sequences had the following parameters: TR = 2400 ms.; TE = 2.22 ms.; Slice thickness = 0.8 mm.; Slices = 208; Voxel size = 0.8 mm × 0.8 mm × 0.8 mm; Acquisition time = 6 min 38 s; GRAPPA = 2. T2-weighted sequences had the following parameters: TR = 3200 ms.; TE = 563 ms.; Slice thickness = 0.8 mm.; Slices = 208; Voxel size = 0.8 mm × 0.8 mm × 0.8 mm; Acquisition time = 5 min 57 s; GRAPPA = 2. Following, resting state fMRI (rs-fMRI) with a multiband echo planar imaging (EPI) sequence supplemented with a single-band EPI image and 2 spin-echo field maps with opposing phase-encoding directions for distortion correction was collected. During this sequence, participants were instructed to clear their minds, keep their eyes open, and look directly at a stationary fixation cross. The rs-fMRI scan parameters were: TR = 800 ms.; TE = 37 ms.; flip angle = 52°, Slice thickness = 2.0 mm.; Slices = 72; Voxel size = 2.0 mm × 2.0 mm × 2.0 mm; Multiband acceleration factor = 8; Acquisition time = 15 min and 20 s.

Anatomical MRI data were first converted from DICOM to NIFTI format using dcm2niix software (Li et al., 2016). Following data conversion, MRI data were processed through a standardized pipeline adapted from the HCP-A (Glasser et al., 2013). Briefly, the HCP-A pipeline for anatomical data includes brain tissue segmentation, reconstruction of cortical models, removal of non-brain tissue, non-linear registration to template space (MNI2009c), identification of gray/white matter boundaries, cortical thickness calculation, and smoothing of cortical surface reconstructions (Dale et al., 1999; Fischl et al., 1999; Fischl and Dale, 2000).

Preprocessing of rs-fMRI images with HCP-A pipelines included: distortion correction, volumetric co-registration of EPI volumes to single-band EPI reference images, and registration from EPI to individual anatomical space (Glasser et al., 2013; Harms et al., 2018). After preprocessing, Analysis of Functional NeuroImages (AFNI) software tools were used for additional processing (Cox, 1996). Each functional run was bandpass filtered to retain frequencies between 0.008 Hz and 0.09 Hz. Volumes containing significant motion or large signal outliers (≥10% of voxels) were censored during regression, bandpass filtering, and later analysis. Bandpass filtering was carried out during a single unified regression step (Fox et al., 2005).

#### 2.3.4 Modularity analysis

Functional scans were further processed to measure modularity using Connectome Workbench software from the Connectome Coordination Facility (Marcus et al., 2011). Scans were first parcellated using the Gordon 333 parcellation, a map of the intrinsic networks of the brain generated from a large dataset of functional MRI scans (Gordon et al., 2016) that parcels the brain into discrete units and assigns them to functional networks. Following parcellation, the fMRI signal of voxels within parcels were averaged to determine the mean timeseries of each parcel. Pearson's *r* correlations were then calculated for each pair of parcels and their respective timeseries. The resulting correlations were compiled into a correlation matrix with each parcel grouped into the intrinsic functional network as specified by the Gordon 333 parcellation. We then applied the correlation matrix to calculate whole brain modularity applying multiple thresholds of correlation strength to generate datasets containing only the 80% to 98% of strongest correlations in steps of 2%. The thresholded datasets were then used to calculate their respective Newman modularity values through the Brain Connectivity bctpy tool (Rubinov and Sporns, 2010). Modularity provides a simple scalar representation of the segregation of the functional networks of the brain. It is a unitless measure ranging from −1.0 to 1.0 such that higher values indicate greater “modularity” reflecting greater segregation of functional networks (Cohen and D'Esposito, 2016).

#### 2.3.5 Cognitive testing

Cognitive function, operationalized as executive functions and working memory, was measured using standardized cognitive tasks administered via the BrainBaseline iPad application (Clinical ink, Horsham, PA, USA) (Lee et al., 2012). These specific domains of cognition were chosen because of their established associations with CACD and well-documented improvement with exercise in older adults (Northey et al., 2018). The BrainBaseline application has been validated across age groups (Lee et al., 2012) and utilized in numerous clinical populations including older adults (Clark et al., 2017), cancer survivors (Ehlers et al., 2017), and individuals diagnosed with HIV (Rubin et al., 2021). Full details of these cognitive tasks have been published previously (Ehlers et al., 2017).

Executive functions were operationalized as incongruent reaction time on the Stroop task and total time to completion for Trails-B. For the Stroop task (Stroop, 1935), participants were presented with words displayed in varying colored text. Participants were then asked to identify the color of the word in neutral (e.g., the word “cat” presented in the color red), congruent (e.g., the word “blue” presented in blue text), or incongruent (e.g., the word “blue” presented in red text) conditions. The Trails-B (Tombaugh, 2004) task required participants to use their finger to draw lines between alternating numbers and letters in ascending order (e.g., 1, A, 2, B, 3, C, etc.).

Working memory was operationalized as accuracy and reaction time on the N-back task in the 2-back condition and accuracy and reaction time on the SPWM task in the set-size 3 condition. The N-back (Owen et al., 2005) task consisted of a single stream of letters presented in succession. Participants were asked to determine whether the letter displayed matched the letter shown two items before (hence, the “2-back” condition). During SPWM (Awh et al., 1998), participants were presented with an image containing 3 black dots. After a brief delay, participants were presented with an image of a single red dot and asked to identify whether the location of the red dot matched the location of one of the 3 previously shown black dots.

### 2.4 Data analysis

All data analyses were exploratory and designed to calculate preliminary effects estimates for hypothesis generation for a future, larger study. Based on the small sample size, whole-brain modularity, MVPA, and cognitive performance are reported descriptively by individual. However, to provide preliminary estimates of effect, between group differences in these outcomes were also evaluated using Cohen's *d* effect estimates. Effect sizes of ≥0.8, ≥0.5, ≥0.2 were considered large, medium, and small, respectively (Lakens, 2013). We also conducted an exploratory one-tailed, paired *t*-tests analysis within groups because prior work has shown that modularity increases after an exercise intervention (Burdette et al., 2010; Voss et al., 2010, 2020). Specifically, we evaluated change in modularity within groups from baseline to 3-month follow-up from the 80%-98% thresholds (Voss et al., 2010, 2016, 2020). Modularity analyses were repeated across thresholds to binarize regional connectivity (n.b. these thresholds play a different role than statistical thresholds used for significance testing) (Bullmore and Sporns, 2009; Power et al., 2011; Gordon et al., 2016). Measuring network modularity at multiple thresholds ensured that any isolated, spurious associations were not improperly assumed to be robust effects. Rather, observing statistical significance and/or directionality of associations at multiple thresholds would increase confidence that such effects were reliable within the dataset being analyzed.

Bivariate Spearman's correlations were calculated to explore associations between (1) change in MVPA and change in modularity and (2) change in modularity and change in cognitive performance from baseline to 3-month follow-up. Accuracy and reaction time data were considered as separate outcomes because they are inversely related. Data were analyzed using SPSS version 27 (IBM Corp., Armonk, NY, USA) and R (R Core Team).

## 3 Results

### 3.1 Sample characteristics

Women in this study were, on average, Caucasian, overweight, older adults who had been diagnosed with early-stage breast cancer, had completed primary treatment within 1 year prior to enrollment, and were prescribed adjuvant hormonal therapy for their cancer. All participants were currently prescribed hormonal therapy, half received radiation therapy, and only 3 BCS (all usual care) received chemotherapy. Full details of the sample are presented in Table 1.

**Table 1:** Participant characteristics.

	**Exercise (*****n*** = **4)**		**Usual Care (*****n*** = **6)**		**Total (*****N*** = **10)**	
	**M**	**±SD^a^**	**M**	**±SD**	**M**	**±SD**
	***n***	**(%)**	***n***	**(%)**	***n***	**(%)**
**Demographics**						
Age (years)	66.0	±4.08	65.8	±12.11	65.9	±9.33
Bachelor's Degree	2	(50.0)	2	(33.3)	4	(40.0)
Income ≥ $75, 000 per year	3	(75.0)	1	(16.7)	6	(78.9)
Employed full-time	1	(25.0)	1	(16.7)	2	(20.0)
White	4	(100.0)	5	(83.8)	9	(90.0)
Married	3	(75.0)	2	(33.3)	5	(50.0)
Body mass index (kg/m^2^)	27.2	±5.54	30.7	±3.67	29.3	±4.57
**Clinical**						
**Cancer Stage**						
1	4	(100.0)	5	(83.8)	9	(90.0)
2	0	(0.0)	1	(16.7)	1	(10.0)
Months since diagnosis	8.5	±6.35	15.5	±7.48	12.7	±7.59
Months since last treatment	7.5	±5.74	12.0	±8.07	10.2	±7.25
Radiation only	3	(75.0)	2	(33.3)	5	(50.0)
Chemotherapy and radiation	0	(0.0)	3	(50.0)	3	(30.0)
Hormonal therapy	4	(100.0)	6	(100.0)	10	(100.0)
Hormonal therapy (months)	7.3	±4.73	8.8	±6.27	8.3	±5.55
Diagnosed with Depression	2	(50.0)	2	(33.3)	4	(40.0)

^*a*^Mean, Standard Deviation.

### 3.2 Effect sizes: modularity, MVPA, cognition

The magnitude of change in modularity between groups yielded small to medium effect sizes across thresholds [range: *d* = 0.23 (80^%^ threshold), *d* = 0.67 (96% threshold)]. Individual and aggregate values at the 80% threshold are reported in Table 2, with aggregate values on all thresholds available in Supplementary Table 1. BCS randomized to aerobic exercise increased daily MVPA by 2.3 min while those randomized to usual care decreased daily MVPA by 9.2 min per day (Table 2). Change in MVPA between groups yielded a medium effect size (*d* = 0.60). The magnitude of change in cognitive performance varied across tasks, with changes in Stroop and Trails-B (i.e., executive function) favoring the control group and changes in N-Back and SPWM (i.e., working memory) favoring the aerobic exercise group (Table 2)

**Table 2:** Brain modularity, physical activity, and cognitive function between exercise and usual care participants.

**fMRI**	**Modularity score**			**Modularity score**		
**Usual care group**	**Baseline**	**Month 3**	**Exercise group**	**Baseline**	**Month 3**	**ES**
UCG (M ± SD)	0.1626 ± 0.012	0.1704 ± 0.016	EG (M ± SD)	0.1611 ± 0.009	0.1707 ± 0.006	0.23
UC-1	0.1666	0.1711	EG-1	0.1656	0.1672	
UC-2	0.1630	0.1602	EG-2	0.1650	0.1764	
UC-3	0.1668	0.1756	EG-3	0.1658	0.1749	
UC-4	0.1675	0.1657	EG-4	0.1587	0.1644	
UC-5	0.1733	0.1980				
UC-6	0.1384	0.1519				
**Physical activity**	**MVPA (min)**			**MVPA (min)**		
**Usual care group**	**Baseline**	**Month 3**	**Exercise Group**	**Baseline**	**Month 3**	**ES**
UCG (M ± SD)	20.3 ± 15.7	11.1 ± 10.1	EG (M ± SD)	23.3 ± 6.6	25.7 ± 23.2	0.60
UC-1	13.7	25.9	EG-1	18.4	6.2	
UC-2	34.1	6.9	EG-2	20.0	11.4	
UC-3	39.7	N/A	EG-3	33.0	57.8	
UC-4	N/A	N/A	EG-4	22.0	27.3	
UC-5	4.6	3.4				
UC-6	9.1	8.2				
**Cognitive function**						
**Stroop**	**Incongruent RT**			**Incongruent RT**		
**Usual care group**	**Baseline**	**Month 3**	**Exercise Group**	**Baseline**	**Month 3**	**ES**
UCG (M ± SD)	1358.5 ± 278.9	1231.5 ± 216.7	EG (M ± SD)	1154.8 ± 191.5	1090.0 ± 144.0	−0.39
UC-1	1101.0	1231.0	EG-1	1311.7	1284.5	
UC-2	1654.2	1587.5	EG-2	883.4	1076.9	
UC-3	1254.7	1027.7	EG-3	1262.9	1061.5	
UC-4	1762.2	1278.1	EG-4	1161.3	937.1	
UC-5	1236.5	1100.9				
UC-6	1143.0	1063.9				
**Trails-B**	**Total time (s)**			**Total time (s)**		**ES**
UCG (M ± SD)	90.6 ± 30.3	73.1 ± 12.8	EG (M ± SD)	55.8 ± 7.5	56.5 ± 20.0	−0.77
UC-1	72.0	78.1	EG-1	61.1	84.4	
UC-2	83.6	75.1	EG-2	61.6	38.0	
UC-3	79.9	50.9	EG-3	45.4	56.3	
UC-4	58.6	70.7	EG-4	54.9	47.4	
UC-5	143.5	73.4				
UC-6	106.2	90.1				
**N-Back**	**2-Back accuracy**			**2-Back accuracy**		**ES**
UCG (M ± SD)	0.63 ±0.2	0.73 ±0.14	EG (M ± SD)	0.61 ±0.23	0.85 ±0.09	0.74
UC-1	0.28	0.56	EG-1	0.67	0.83	
UC-2	0.72	0.83	EG-2	0.33	0.89	
UC-3	0.83	0.78	EG-3	0.56	0.72	
UC-4	0.72	0.56	EG-4	0.89	0.94	
UC-5	0.50	0.83				
UC-6	0.72	0.83				
**N-Back**	**2-Back RT (ms)** ^*^			**2-Back RT (ms)** ^*^		**ES**
UCG (M ± SD)	1146.4 ± 100.5	1153.1 ± 26.2	EG (M ± SD)	1069.8 ± 273.4	957.1 ± 94.2	0.87
UC-1	984.6	1138.9	EG-1	1246.9	1016.2	
UC-2	1234.0	1162.4	EG-2	726.3	819.6	
UC-3	1126.7	1121.5	EG-3	1327.8	972.5	
UC-4	1259.3	1137.1	EG-4	978.5	1020.1	
UC-5	1095.8	1162.9				
UC-6	1177.9	1195.6				
**SPWM**	**SS3 accuracy**			**SS3 accuracy**		**ES**
UCG (M ± SD)	0.74 ± 0.1	0.81 ± 0.1	EG (M ± SD)	0.82 ± 0.08	0.93 ± 0.07	0.49
UC-1	0.77	0.70	EG-1	0.77	0.83	
UC-2	0.67	0.67	EG-2	0.90	1.0	
UC-3	0.80	0.93	EG-3	0.73	0.93	
UC-4	0.87	0.87	EG-4	0.87	0.93	
UC-5	0.77	0.87				
UC-6	0.57	0.80				
**SPWM**	**SS3 RT (ms)**			**SS3 RT (ms)**		**ES**
UCG (M ± SD)	991.3 ± 159.2	997.9 ± 92.9	EG (M ± SD)	887.4 ± 126.2	880.7 ± 161.3	0.06
UC-1	925.0	1018.2	EG-1	951.7	830.9	
UC-2	959.2	898.8	EG-2	731.5	680.3	
UC-3	953.3	1001.1	EG-3	1020.3	960.5	
UC-4	1174.4	1099.4	EG-4	846.0	1051.0	
UC-5	1174.3	1009.4				
UC-6	761.3	840.3				

Data presented include mean modularity by group and individual participant modularity. Modularity thresholded at a representative 80th percentile of connections. Positive effect sizes indicate benefits favoring the exercise group. M, Mean; SD, standard deviation; ES, effect size expressed as Cohen's d; MVPA, moderate-to-vigorous physical activity; RT, reaction time; SPWM, spatial working memory; SS3, set size 3; fMRI, functional magnetic resonance imaging; UCG, usual care group; EG, exercise group.

### 3.3 Modularity: exploratory *t*-tests

**Figure 2:**
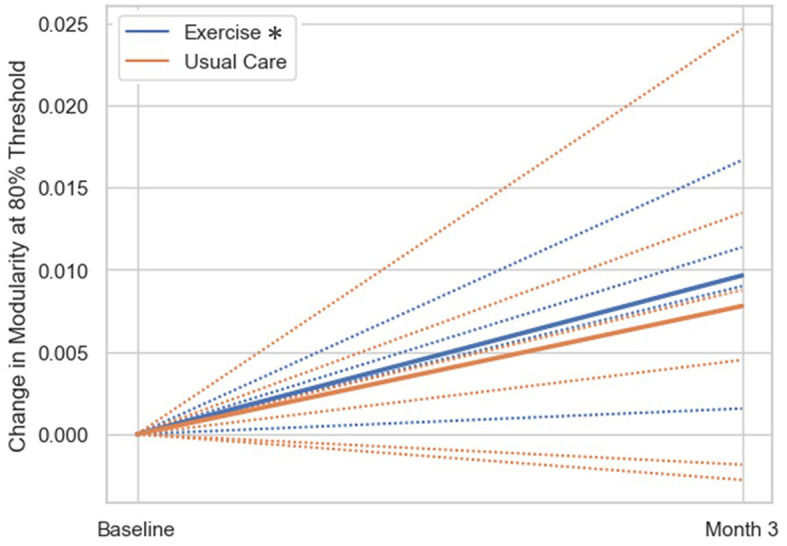
Change in modularity scores from baseline to 3-month follow-up by group. Change in modularity at 80% threshold (group mean = solid line, individual subjects = dashed lines). The exercise group had significantly increased modularity at the 3-month follow up compared to baseline (*p < 0.05), while the usual care group did not show significant changes in modularity from baseline to 3-month follow-up.

**Figure 3:**
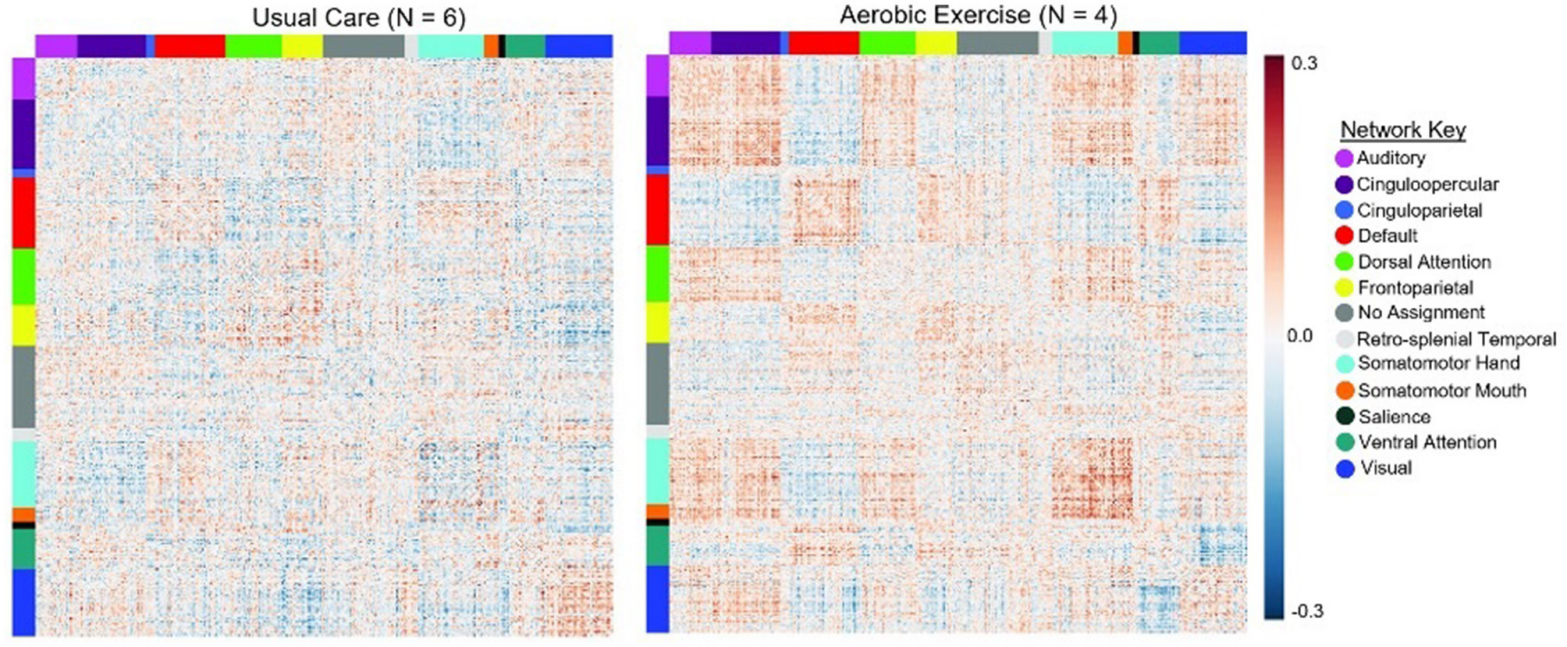
Average change in network correlation from baseline to 3-month follow-up in the usual care and exercise groups. Change in modularity from baseline to 3-month follow up is displayed at a representative 80% threshold in the UC and AE groups. This qualitative representation of changes within and between brain networks demonstrates that in the AE group there was increased within-network connectivity and decreased between-network connectivity, while these patterns were attenuated in the UC group.

The exploratory within-group analysis revealed statistically significant positive changes in modularity from baseline to 3-month follow-up in the aerobic exercise group at 80%, 82%, and 84% thresholds (Figure 2; Supplementary Table 1). There was no significant change in modularity between baseline and 3-month follow up in the usual care group at any threshold (Supplementary Table 1). The individual data highlight how patients in the exercise group demonstrated an increase in modularity, while the usual care group had more variable changes in modularity from baseline to 3-month follow-up. The difference in network correlation from baseline to 3-month follow-up is represented by a cross correlation matrix for each group in Figure 3. This qualitative representation of changes within and between brain networks depicts increased within-network connectivity and decreased between-network connectivity in the aerobic exercise group. While the usual care group also displays some decreases in between-network connectivity, the overall patterns were attenuated compared with the aerobic exercise group.

### 3.4 Associations among physical activity, modularity, and cognitive function

Correlation analyses revealed a weak, positive correlation between change in MVPA and change in 80% threshold modularity; however, it did not achieve statistical significance (*r* = 0.36, *p* = 0.39). There were no statistically significant correlations between change in modularity and change in cognitive performance outcomes (all *p* > 0.09; Table 3). For completeness, we describe the observed, non-significant patterns in the remainder of this section, with the caveat that larger samples sizes will likely be required for rigorous evaluation. Directionality of the associations indicated that increased modularity was associated with small improvements in Stroop incongruent reaction time, Trails-B total time, N-back 2-Back accuracy, and SWPM accuracy. Correlations between change in modularity and cognitive outcomes across all thresholds are included in Supplementary Table 2.

**Table 3:** Correlations of change in whole-brain modularity with MVPA and cognitive performance.

	**Modularity**	
	***r*** **(*****p*****)**	
MVPA	0.36	(0.39)
Stroop		
Incongruent RT	−0.44	(0.88)
Trails-B		
Total Time	−0.56	(0.09)
N-Back		
2-Back accuracy	0.32	(0.37)
2-Back RT	0.47	(0.17)
SPWM		
SS3 accuracy	0.58	(0.28)
SS3 RT	0.25	(0.49)

r, Spearman's rho; MVPA, moderate-to-vigorous physical activity; RT, reaction time; SPWM, spatial working memory; SS3, set size 3. Modularity presented as score at 80th percentile of correlation strength. Beneficial associations are indicated by negative correlations for timed outcomes and positive correlations for accuracy and MVPA outcomes.

## 4 Discussion

The primary objective of this exploratory study was to examine the effects of a 12-week aerobic exercise program on whole-brain modularity in post-menopausal BCS. Major findings suggest modularity may be a novel brain biomarker warranting further exploration in exercise oncology. The exploratory modularity analysis revealed that BCS in the aerobic exercise group demonstrated improvements in modularity from baseline to 3-month follow-up, while the usual care group did not. Despite the small sample size, these preliminary results revealed that the neuroimaging approach was sensitive to changes in brain network organization after only 12 weeks of aerobic exercise training. However, change in modularity was not significantly correlated with changes in MVPA or cognitive performance. How or whether these changes are reliably associated with MVPA and cognitive performance remains unclear, but these associations warrant future research.

Whole brain modularity has been used previously to describe changes in functional connectivity associated with aging and disease (Chan et al., 2014; Aboud et al., 2019) as well as to predict cognitive improvements after exercise and cognitive training interventions (Baniqued et al., 2018, 2019). Analyses in the present study revealed that between group changes in modularity favored the aerobic exercise group with a small to medium effect size. The exploratory *t*-tests demonstrated that even a short, moderate-intensity aerobic exercise intervention may yield positive changes in brain network functional organization, and qualitative observations were consistent with modularity increases observed only in the aerobic exercise group. These findings are notable because they replicate evidence of modularity changes in aging adults after participation in an exercise intervention (Burdette et al., 2010; Baniqued et al., 2019). Broadly, evidence from computational models have demonstrated that modular networks are able to adapt more efficiently to new environments, (Kashtan and Alon, 2005; Tosh and McNally, 2015), and evidence from MRI studies have found that optimally modular networks require lower functional demand to perform cognitive tasks (Stevens et al., 2012; Fornito et al., 2015; Baniqued et al., 2018). In contrast, aging-related disruptions in brain modularity are associated with lower cognitive efficiency and hypothesized to occur prior to anatomical atrophy and behavioral cognitive impairment (Onoda and Yamaguchi, 2013). Taken together with findings presented in this study, it is plausible that exercise interventions could serve as an important first step in ameliorating cognitive declines in BCS through the improvement of brain modularity.

In contrast to previous research (Galiano-Castillo et al., 2017; Campbell et al., 2018; Hartman et al., 2018), there were no associations between change in modularity and change in cognitive performance, despite some correlations trending toward significance and being of moderate strength. This may be at least partially explained by trends observed in age-related decline in which stronger changes observed in modularity preceded behavioral changes in cognitive performance (Onoda and Yamaguchi, 2013). Additionally, previously published MRI data have suggested that BCS exhibit compensatory brain activity during cognitive tasks characteristic of network dysfunction (Kesler et al., 2017; Phillips et al., 2022). Therefore, biomarkers of functional organization, such as brain modularity, may be useful for identifying both subclinical declines in brain health and intervention-related improvements in studies of CACD. While cognitive training intervention studies have found improvements in task performance after only 5 to 8 weeks, aerobic exercise-related cognitive gains may take longer to manifest. Indeed, improvements in executive functions observed by Baniqued et al. (2018) were the result of a 6-month exercise program, that is, twice the length of the program in this study, and was conducted in healthy older adults without known memory impairments. Furthermore, findings from another previous study in older adults only found favorable effects on functional connectivity and executive functions after 12-months of exercise training, with no change observed at the interim 6-month follow-up (Voss et al., 2010). Amid promising, but inconsistent, evidence on exercise's neurocognitive benefits amid cancer (Campbell et al., 2019), it is unclear whether the trajectory of functional connectivity and cognitive responses to exercise training in a cancer population would mirror those previously reported in aging. Nevertheless, modularity should be considered in future studies of CACD because it may provide a metric of intervention-related cognitive improvements that, based on the present findings, may be modified over even short periods of time.

Of further interest was the weak, non-significant correlation between modularity and MVPA. Findings from previous imaging studies in aging have indicated that it may be changes in cardiorespiratory fitness, not minutes of MVPA, that drive neural and cognitive changes associated with exercise interventions (Chaddock-Heyman et al., 2015; Oberlin et al., 2016; Voss et al., 2016; Lesnovskaya et al., 2023). However, while cross-sectional studies have observed positive associations between fitness and neural biomarkers in BCS (Chaddock-Heyman et al., 2015; Lesnovskaya et al., 2023), fitness-related improvements have not yet been supported by emergent data (Koevoets et al., 2023).

### 4.1 Strengths and limitations

This study was the one of the first to examine the effects of aerobic exercise training on neural outcomes in cancer survivors and, to our knowledge, the first to investigate brain network modularity (Koevoets et al., 2023). Furthermore, it employed a rigorous fMRI protocol to evaluate a novel brain biomarker with implications for successful brain aging and provided promising preliminary results. It is, however, limited in its conclusions due to the pilot design and small sample size. As such, results should be interpreted with caution and be considered as hypothesis generating. It is possible that other factors influenced brain changes other than the exercise intervention that were not observable in this analysis. Specifically, BCS with a history of chemotherapy treatment were not evenly distributed between treatment groups, which may have biased results favoring the intervention. As there was only a weak correlation between change in MVPA and change in modularity, it is unclear to what extent other unknown factors related to the intervention, other than increasing physical activity, such as improved fitness or improved psychosocial function, were associated with changes in modularity. Larger studies are needed to fully understand the effects of exercise interventions on brain network organization. The sample was also primarily composed of white BCS with early-stage cancer who had not received chemotherapy and that were not required to have cognitive complaints to be eligible for participation, which limits the generalizability of results to the broader breast cancer population.

Modularity as a biomarker for CACD has many strengths, but it is not without limitations. Parcellating functional data to split the brain into discrete functional units allows calculations of modularity and other connectomic measures; however, it relies on a large normative sample to generate a parcellation (Gordon et al., 2016). It is therefore possible that some features unique to individuals or non-healthy populations are lost. For example, the Gordon 333 parcellation was generated utilizing functional MRI data from healthy adults (Gordon et al., 2016). This parcellation is well-validated and widely used, but the development of a BCS-specific functional parcellation might benefit future efforts. Additionally, as modularity is a whole brain metric, it does not assess changes within specific networks. Identifying network-specific changes associated with CACD and exercise interventions is a key future direction for this line of research.

### 4.2 Conclusion

The primary objective was to evaluate modularity as a biomarker of exercise intervention-related changes in brain health. Findings indicated that the 12-week exercise intervention was associated with a medium effect on daily MVPA and brain modularity. There was a weak correlation between change in MVPA and change in modularity; the small sample size and limited power may have weakened this association. The exploratory analysis revealed that at the 80%-84% thresholds, BCS in the aerobic exercise group had significantly higher whole-brain modularity at the 3-month follow-up, and the directionality, albeit not the statistical significance, of the effect was consistent across all thresholds. Given that the direction of the associations between modularity, physical activity, and cognition were in the expected direction, results from this study indicate that further investigation of these relationships is warranted.

## Data availability statement

The raw data supporting the conclusions of this article will be made available by the authors, without undue reservation.

## Ethics statement

The studies involving humans were approved by the University of Nebraska Medical Center Institutional Review Board. The studies were conducted in accordance with the local legislation and institutional requirements. The participants provided their written informed consent to participate in this study.

## Author contributions

LP: Conceptualization, Data curation, Formal analysis, Investigation, Methodology, Project administration, Visualization, Writing – original draft, Writing – review & editing. AH-W: Conceptualization, Methodology, Writing – original draft, Writing – review & editing. CP: Formal analysis, Methodology, Writing – review & editing. AB: Writing – review & editing. ER: Writing – review & editing. DW: Methodology, Supervision, Writing – review & editing. DE: Conceptualization, Data curation, Funding acquisition, Investigation, Methodology, Project administration, Supervision, Writing – review & editing.
