# Supplementary material for: Modifying modularity: aerobic exercise improves functional connectivity in breast cancer survivors
Source: Front Cognit. 2024 Feb 2;3:1323438. doi: 10.3389/fcogn.2024.1323438 (PMC13281212; doi:10.3389/fcogn.2024.1323438)
Supplement: Supplementary file 1 [file Table_1.DOCX]

| **Supplemental Table 1.** Modularity values across thresholds at baseline and at 3-month follow-up. | | | | | | | | | | | | | | |
| --- | --- | --- | --- | --- | --- | --- | --- | --- | --- | --- | --- | --- | --- | --- |
|  | **Exercise (N = 4)** | | | | | | | **Usual Care (N = 6)** | | | | | | |
|  | **Baseline** | | **Month 3** | |  | | | **Baseline** | | **Month 3** | |  | | |
| **Modularity Threshold** | M | ±SD | M | ±SD | *t* | *p* | ES | M | ±SD | M | ±SD | *t* | *p* | ES |
| 80%  82%  84%  86%  88%  90%  92%  94%  96%  98% | 0.1611 | ±0.009 | 0.1707 | ±0.006 | 3.08* | .027 | 1.17 | 0.1626 | ±0.012 | 0.1704 | ±0.016 | 1.85 | .061 | 0.52 |
|  | 0.1739 | ±0.010 | 0.1845 | ±0.007 | 3.10* | .027 | 1.12 | 0.1758 | ±0.015 | 0.1843 | ±0.017 | 2.00 | .051 | 0.52 |
|  | 0.1874 | ±0.011 | 0.2007 | ±0.007 | 2.49* | .044 | 1.30 | 0.1940 | ±0.017 | 0.1992 | ±0.018 | 1.85 | .061 | 0.42 |
|  | 0.2052 | ±0.013 | 0.2183 | ±0.007 | 2.34 | .051 | 1.20 | 0.2074 | ±0.019 | 0.2159 | ±0.021 | 1.66 | .079 | 0.41 |
|  | 0.2231 | ±0.014 | 0.2397 | ±0.006 | 2.18 | .058 | 1.49 | 0.2268 | ±0.022 | 0.2356 | ±0.023 | 1.55 | .091 | 0.39 |
|  | 0.2444 | ±0.015 | 0.2618 | ±0.008 | 1.99 | .070 | 1.45 | 0.2492 | ±0.027 | 0.2585 | ±0.025 | 1.48 | .099 | 0.35 |
|  | 0.2719 | ±0.016 | 0.2877 | ±0.007 | 1.53 | .112 | 1.31 | 0.2786 | ±0.031 | 0.2868 | ±0.030 | 1.73 | .147 | 0.27 |
|  | 0.3047 | ±0.020 | 0.3252 | ±0.010 | 1.39 | .129 | 1.37 | 0.3110 | ±0.037 | 0.3228 | ±0.030 | 1.20 | .142 | 0.34 |
|  | 0.3419 | ±0.020 | 0.3694 | ±0.020 | 1.35 | .134 | 1.36 | 0.3584 | ±0.041 | 0.3663 | ±0.037 | 0.72 | .251 | 0.20 |
|  | 0.3978 | ±0.018 | 0.4358 | ±0.045 | 1.23 | .153 | 1.20 | 0.4208 | ±0.046 | 0.4384 | ±0.041 | 1.41 | .109 | 0.40 |
| ***Notes.*** **p* < .05, Mean (M), standard deviation (SD), effect size (ES). | | | | | | | | | | | | | | |

| **Supplemental Table 2.** Correlations of changes in modularity and cognitive performance. | | | | | | | | | | | | | |
| --- | --- | --- | --- | --- | --- | --- | --- | --- | --- | --- | --- | --- | --- |
|  | |  | |  | |  | |  | |  | |  | |
|  | | **Stroop Incongruent RT** | | **Trails-B  Total Time** | | **N-Back 2-Back Accuracy** | | **N-Back 2-Back RT** | | **SPWM SS3 Accuracy** | | **SPWM SS3  RT** | |
| **Modularity Threshold** | | *r* | *(p)* | *r* | *(p)* | *r* | *(p)* | *r* | *(p)* | *r* | *(p)* | *r* | *(p)* |
|  | 80% | -0.44 | (0.88) | -0.56 | (0.09) | 0.32 | (0.37) | 0.47 | (0.17) | 0.58 | (0.08) | 0.25 | (0.49) |
|  | 82% | 0.02 | (0.96) | -0.61 | (0.06) | 0.36 | (0.31) | 0.48 | (0.16) | 0.28 | (0.08) | 0.26 | (0.47) |
|  | 84% | 0.07 | (0.86) | -0.62 | (0.05) | 0.43 | (0.22) | 0.52 | (0.13) | 0.54 | (0.11) | 0.24 | (0.51) |
|  | 86% | 0.03 | (0.93) | -0.76* | (0.01) | 0.35 | (0.33) | 0.59 | (0.07) | 0.50 | (0.14) | 0.29 | (0.43) |
|  | 88% | 0.01 | (0.99) | -0.70 | (0.03) | 0.27 | (0.44) | 0.58 | (0.08) | 0.48 | (0.16) | 0.39 | (0.26) |
|  | 90% | 0.25 | (0.49) | -0.47 | (0.17) | 0.38 | (0.28) | 0.56 | (0.09) | 0.38 | (0.28) | 0.50 | (0.14) |
|  | 92% | 0.25 | (0.49) | -0.56 | (0.09) | 0.38 | (0.28) | 0.55 | (0.10) | 0.28 | (0.44) | 0.35 | (0.33) |
|  | 94% | 0.32 | (0.37) | -0.54 | (0.11) | 0.35 | (0.33) | 0.64* | (0.05) | 0.22 | (0.54) | 0.48 | (0.16) |
|  | 96% | 0.50 | (0.14) | -0.31 | (0.39) | 0.48 | (0.16) | 0.75* | (0.01) | -0.02 | (0.96) | 0.59 | (0.07) |
|  | 98% | 0.39 | (0.26) | -0.27 | (0.45) | 0.45 | (0.19) | 0.76* | (0.01) | -0.15 | (0.67) | 0.47 | (0.17) |
| ***Notes.*** Spearman’s rho (*r*), reaction time (RT), spatial working memory (SPWM), set size 3 (SS3). Modularity presented as score at 80^th^ percentile of correlation strength. Beneficial associations are indicated by negative correlations for timed outcomes and positive correlations for accuracy outcomes.  * *p* < 0.05 | | | | | | | | | | | | | |
